# Lipid-Lowering Medications for Managing Dyslipidemia: A Narrative Review

**DOI:** 10.7759/cureus.65202

**Published:** 2024-07-23

**Authors:** Majed S Alqahtani, Khalid F Alzibali, Abdulaziz Mahdi M Mahdi, Osama Mohammed A Alharbi, Reem Hafiz A Harbi, Hamad Saad M Alkhaldi, Zahrah Ali A Alsayafi, Fatema H Albisher, Murtadha H Buqurayn, Meshal M Alharbi

**Affiliations:** 1 Family Medicine and Diabetes Management, King Fahad Specialized Hospital, Tabuk, SAU; 2 Family Medicine, King Abdulaziz University, Rabigh, SAU; 3 Family Medicine, University of Jeddah, Jeddah, SAU; 4 Family Medicine, Jazan University, Jazan, SAU; 5 General Practice, Imam Mohammed Bin Saud University, Riyadh, SAU; 6 General Practice, Al-Ahsa Health Cluster, Al-Ahsa, SAU; 7 Clinical Pharmacy, Almoosa Specialist Hospital, Alahsa, SAU; 8 General Practice, Ad Diriyah Hospital, Riyadh, SAU

**Keywords:** bempedoic acid, proprotein convertase subtilisin/kexin type 9 inhibitors, bile acid sequestrants, ezetimibe, omega-3 fatty acids, fibrates, statins, dyslipidemia, lipid-lowering medications

## Abstract

Dyslipidemia refers to the change in the normal levels of one or more lipid components in the bloodstream, which include triglycerides (TG), total cholesterol (TC), high-density lipoprotein cholesterol (HDL-C), and low-density lipoprotein cholesterol (LDL-C). Dyslipidemia represents a substantial source of danger for cardiovascular disease (CVD). Effectively managing dyslipidemia involves a thorough strategy that includes changing one's lifestyle and using medications that are specifically designed to target the complex processes involved in lipid metabolism. Lipid-lowering treatments play a crucial role in this approach, providing a wide range of medications that are developed to specifically target different components of dyslipidemia. Statins are the main drug among these medications. Other drugs that are used with statin or as monotherapy include fibrates, omega-3 fatty acids (OM3FAs), ezetimibe, bile acid sequestrants, proprotein convertase subtilisin/kexin type 9 (PCSK9) inhibitors, and bempedoic acid. Using the PubMed database, we reviewed the literature about dyslipidemia, drugs used for treating dyslipidemia, their efficacy parameters, and common adverse events. We also reviewed the international guidelines for treating dyslipidemia and discussed the future of lipid-lowering medications. More trials and experiments are still required to verify the effectiveness of many lipid-lowering drugs and to know their common adverse events to be able to manage them properly.

## Introduction and background

A collection of irregularities associated with the metabolism of plasma lipids and lipoproteins, including low levels of high-density lipoprotein cholesterol (HDL-C), high levels of low-density lipoprotein cholesterol (LDL-C), total cholesterol (TC), and triglycerides (TG), are together referred to as dyslipidemia. There are three possible causes of dyslipidemia: intrinsic, extrinsic, or a mix of environmental and genetic factors. While secondary dyslipidemias result from the correlation of risk factors with other pathologies or environmental factors, initial dyslipidemias are a diverse set of disorders with a genetic, mono, or polygenic etiology [1,2]. Its prevalence is notable, affecting 38.6% of individuals aged 40 years and older [3]. It is considered a predisposing factor for a spectrum of cardiovascular and systemic complications. The risk of cardiovascular disease (CVD) is twice as high in those with dyslipidemia as in those with normal lipid levels [4]. The associations of dyslipidemia with atherosclerosis, endothelial dysfunction, and cardiovascular mortality underscore its clinical significance and necessitate prompt diagnosis, risk assessment, and effective management strategies [5].

Managing dyslipidemia requires a comprehensive approach encompassing diet and lifestyle modifications and pharmacological interventions tailored to address the intricate pathophysiology of lipid metabolism. Lipid-lowering medications are a cornerstone in this therapeutic paradigm, offering a diverse array of pharmacological agents designed to target specific aspects of dyslipidemia [6]. Among these therapeutic agents are statins, which are the first choice in dyslipidemia management and exert potent effects on LDL-C levels [7]. Complementing the LDL-lowering properties of statins, fibrates cause triglyceride level reductions and modest enhancements in HDL-C concentrations [8]. Additionally, ezetimibe disrupts intestinal cholesterol absorption, thereby decreasing LDL-C levels. Bile acid sequestrants, which sequester bile acids within the intestinal lumen, promote fecal excretion of cholesterol and LDL-C reduction [9]. Proprotein convertase subtilisin/kexin type 9 (PCSK9) inhibitors are an important breakthrough in treating dyslipidemia since they lessen the degradation of LDL receptors, allowing for significant drops in LDL-C levels [10]. Finally, omega-3 fatty acids (OM3FAs), known for their triglyceride-lowering properties, modulate hepatic triglyceride synthesis and clearance mechanisms, offering adjunctive therapy to individuals with elevated triglyceride levels [11]. By integrating these pharmacological agents into a comprehensive management strategy, healthcare providers can effectively mitigate cardiovascular risk, optimize lipid profiles, and ultimately improve patient outcomes in the management of dyslipidemia.

In this narrative review, we aim to provide a comprehensive view of dyslipidemia management, delving into the pathophysiology of dyslipidemia, the mechanism of lipid-lowering medications, their efficacy, safety profiles, clinical guidelines, and recommendations.

## Review

Methods

We conducted a comprehensive search of the PubMed database to locate scholarly articles published from 2014 to 2024. We used the following search terms: "lipid-lowering," “cholesterol-lowering," “statins," "fibrates," “PCSK9 inhibitors," "ezetimibe," “bile acid sequestrants," “bempedoic acid," “omega-3 fatty acids," and "hyperlipidemia." We focused on the clinical trials and systematic reviews discussing the comparative effectiveness of different lipid-lowering agents and provided insights into their safety considerations, drug interactions, and contraindications.

Pathophysiology of dyslipidemia

The primary cause of the atherosclerosis that leads to CVD is dyslipidemia. Oxidation of cholesterol associated with dyslipidemia expedites the description of intercellular adhesion molecule (ICAM)-1 for monocyte adhesion and endothelial selectin (E-selectin). A sequence in monocyte inflow and cytokine production follows this. Monocytes undergo macrophage development and manufacture the protein monocyte chemoattractant protein (MCP)-1 in preparation for more monocyte inflow. Furthermore, monocytes emit oxidizing substances that encourage the oxidation of cholesterol and generate cytokines such as interleukin (IL)-6. Macrophages transform into foam cells that are deposited on the walls of blood vessels after consuming oxidized cholesterol. Atherosclerosis and plaque formation are the results of this process. In this approach, dyslipidemia increases the risk of atherosclerosis and CVD [12-17]. The movement and retention of plasma LDL through the endothelial cell layer and into the extracellular matrix of the subendothelial region cause atherosclerotic lesions. Low-density lipoprotein undergoes nonenzymatic glycation and oxidation in the arterial wall to undergo chemical modification. This moderately oxidized LDL attracts monocytes to the arterial wall. Afterward, these monocytes develop into macrophages, which raise LDL oxidation. These atherosclerotic plaques undergo repeated damage and repair, which stimulates the formation of a fibrous cap. This cap protects the underlying core of lipids, collagen, calcium, and inflammatory cells like T lymphocytes. It is essential to maintain the fibrous plaque to prevent plaque rupture and coronary thrombosis [18,19]. 

Because oxidative stress is directly linked to endothelial dysfunction and the stimulation of the vascular inflammatory response through the increased production of reactive oxygen species (ROS), it is one of the fundamental pathogenetic processes of atherosclerosis. Rapid ROS generation is associated with common illnesses like smoking, diabetes, high blood pressure, and hypercholesterolemia, which are all known to be cardiovascular risk factors that predispose to atherosclerosis. Atherosclerosis is another name for the inflammatory disease of the major and medium arteries. Cytokines have a major role in the pathogenesis of atherosclerosis since they are present at every stage of the disease. Pro-atherogenic cytokines generated by natural killer cells, vascular smooth muscle cells, lymphocytes, and macrophages include IL-1, IL-6, and tumor necrosis factor-α (TNF-α). Both TNF-α and IL-1 promote the formation of adhesion molecules, cytokines, and the migration and mitogenesis of vascular smooth muscle and endothelial cells on the arterial wall during the atherosclerotic process [20-23].

As a major risk factor for CVD, dyslipidemia contributes to an estimated 4.4 million global deaths annually worldwide [7]. Evidence from the Cholesterol Treatment Trialists’ Collaborators reveals that a reduction of 1.0 mmol/l in LDL-C yields a 9% decrease in all-cause mortality and a 25% reduction in major vascular events, even among individuals with low risk [24]. Notably, a strong and incremental correlation exists between TC and LDL-C levels and mortality due to CVD. The intricate interplay among cardiovascular risk factors, inclusive of dyslipidemia, endothelial dysfunction, and atherosclerosis, significantly influences the onset of CVD [25].

Moreover, dyslipidemia exerts profound effects extending beyond cardiovascular health and is associated with metabolic syndrome, diabetes mellitus, and nonalcoholic fatty liver disease, thereby heightening the risk of systemic complications such as insulin resistance, hepatic steatosis, and pancreatic dysfunction. Additionally, dyslipidemia emerges as a predisposing factor for the progression of chronic kidney disease (CKD) [25].

Understanding the clinical relevance of dyslipidemia emphasizes how urgently it must be diagnosed, a thorough risk assessment must be conducted, and efficient management strategies must be implemented to avoid its negative effects. Healthcare professionals may significantly enhance the achievement of cholesterol goals and minimize the incidence of CVD by combining lipid-lowering drugs with lifestyle changes [26].

Role of lipid-lowering agents in managing dyslipidemia

Lipid-lowering medications constitute an integral component of the comprehensive management of dyslipidemia and serve as pivotal tools for attenuating cardiovascular risk and ameliorating adverse lipid profiles [6]. This therapeutic paradigm encompasses various pharmacological agents, each tailored to target distinct aspects of lipid metabolism and atherogenic lipoprotein regulation.

The beginning of cholesterol-lowering pharmacotherapy in modern history traces back to 1972, when Dr. Akira Endo identified compactin, an active compound inhibiting the biosynthesis of cholesterol, at Sankyo Pharmaceutical Company [27]. Statins, introduced in 1987, have marked a significant milestone in the treatment of atherosclerotic CVD [7]. Although high-intensity statin therapy demonstrates notable efficacy in reducing CVD incidence, its use alone often falls short of achieving the recommended reductions for high- and very high-risk patients according to current guidelines [27]. Consequently, various nonstatin medications featuring complementary mechanisms of action to statins have emerged, including, monoclonal antibodies targeting PCSK9 inhibitors, which are monoclonal antibodies, ezetimibe, bempedoic acid, inclisiran, and evinacumab. 

Ezetimibe was the first agent identified to inhibit the absorption of intestinal cholesterol. It has been a significant therapeutic option for dyslipidemia since the advent of statins [9]. Its approval by the US Food and Drug Administration in October 2002 marked a pivotal milestone. Subsequently, in the following year, the relationship between dyslipidemia and PCSK9 mutations was discovered, drawing attention to a new trend in cholesterol-lowering drugs [10,28]. Small-interfering RNA approaches and monoclonal antibodies are now targeting PCSK9, newly developed and clinically approved in 2015 and 2022, respectively [29]. 

Pharmacotherapy needs to be carefully considered in light of the CVD risk assessment. Effective pharmacological management of patients with dyslipidemia requires proper clinical assessment as well as a comparison of the benefits of pharmacological intervention with the risks of initiating potentially lifelong drug therapy for each patient. The selection of an optimal lipid-lowering medication hinges on a nuanced consideration of patient-specific factors, lipid profile characteristics, cardiovascular risk stratification, therapeutic goals, age, and severity [30,31]. The patient should be referred for a hospital consultation if pharmaceutical therapy is indicated [31].

Effective pharmacological management of patients with dyslipidemia requires proper clinical assessment as well as a comparison of the benefits of pharmacological intervention with the risks of initiating potentially lifelong drug therapy for each patient. The selection of an optimal lipid-lowering medication hinges on a nuanced consideration of patient-specific factors, lipid profile characteristics, cardiovascular risk stratification, and therapeutic goals.

Statins

Statins inhibit the 3-hydroxy-3-methylglutaryl-coenzyme A (HMG-CoA) reductase (HMGCR) enzyme, which is frequently prescribed to lower the concentrations of cholesterol and LDL in the bloodstream [32]. Akira Endo and his team obtained three active metabolites from *Penicillium citrinum*; the most effective one was ML-236B, which later became the most important substrate in the production of statin drugs. It was named Compactin (mevastatin) [33]. Statins inhibit HMG-CoA from being converted to mevalonate by connecting to the HMGCR enzyme competitively and reversibly [33]. This enzyme restricts endogenous cholesterol synthesis with decreased intracellular cholesterol concentration and quicker LDL clearance. This is the rate-limiting step in hepatic cholesterol secretion [25,34]. Beyond their primary role in the reduction of LDL-C, statins have multi-functioning effects, including endothelial function enhancement and anti-inflammatory properties, contributing to their comprehensive cardiovascular benefits [35,36]. The main effects of statins are a reduction of LDL and, to a lesser degree, a reduction of triglycerides and an elevation of HDL. Moreover, statins can stabilize atherosclerotic plaques and have anti-inflammatory and anti-thrombotic qualities [37].

Statins have the advantage that they are very selective HMGCRs. So at the site of their action, they nearly don’t interfere with other drugs, but they are subject to other types of drug interactions [38]. They are metabolized in the liver by cytochrome p450 isoenzymes (CYPs). So, drugs that cause the induction of CYPs lower the plasma level of statins, and vice versa [38]. Pitavastatin is the only statin nearly unaffected by this interaction, as it is protected from being metabolized by CYPs [38]. 

When treating dyslipidemia, statins continue to be the first line of treatment. Regardless of baseline lipid levels, the American Diabetes Association (ADA) recommends using statins in patients who have CVD and DM or individuals over 40 with one or more CVD risk factors, such as smoking or a family history of the disease such as dyslipidemia, hypertension, or albuminuria. For diabetic individuals under 40 with several CVD risk factors or LDL >100 mg/dl (82.6 mmol/l) despite lifestyle modification, statin therapy is also advised [39]. A high dose of statins is also recommended for treating patients with heterozygous familial hypercholesterolemia (HeFH) [40]. The most commonly prescribed are pravastatin (PRA) or rosuvastatin (ROS) (over the age of eight), with atorvastatin (ATO), simvastatin (SIM), or lovastatin (LOV) being advised for those over the age of 10 [41]. As the activity of the key enzyme HMGCR is higher at midnight, statins should be taken at bedtime except for ATO and ROS, which have a long half-life, so they can be taken at any time [38-41].

To compare the effects of seven different statins on lipid levels in individuals with CVD, dyslipidemia, or DM, a systematic review and network meta-analyses (NMA) of the lipid changes following the administration of specific statins were conducted. Consequently, the analyses employed seven statin monotherapy regimens from 50 trials with a total of 51,956 patients. Statins such as LOV, ROS, ATO, fluvastatin (FLU), PRA, and pitavastatin (PIT) were among them. Rosuvastatin had the highest rating in terms of decreasing LDL-C, with a surface under-cumulated ranking (SUCRA) score of 93.1%. In terms of LDL-C reduction, ROS scores first in LDL-C, ApoB-lowering efficacy, and ApoA1-increasing efficacy, according to the NMAs. In terms of reducing TC and TG and raising HDL-C, LOV and FLU scored highest. This systematic review offers sources and a basis of data for the selection of drugs in statin monotherapies as well as statin combination treatments, with precautions due to the study limitations [42].

The adverse event of statins that occurs most often is the toxicity of skeletal muscles, which might occur more frequently when statins are combined with other drugs that interfere with statins’ pharmacokinetics and lead to their accumulation in patients’ bodies [43]. Patients with chronic liver disease who are treated with statins often have raised the risk of hepatotoxicity as statins undergo metabolism by CYPs [44]. Asymptomatic transaminitis is one of the relatively rare (around 3%) statin adverse events that appear in the first year after the beginning of treatment; it is usually self-limiting, and dose-dependent [44]. Statins are contraindicated for patients who have antibodies against HMGCR. Statins are used in specific patient populations, with considerations summarized in Table 1.

**Table 1:** Summary of statins’ considerations in specific patient populations

Population	Specific considerations
Hepatic impairment [45,46]	When hepatic disease is active, all statins should be avoided. In cases of chronic liver illness, statins with low hepatic metabolism, such as pravastatin and rosuvastatin, are recommended. Patients with metabolic dysfunction-associated steatotic liver disease (MASLD) or nonalcoholic fatty liver disease (NAFLD) benefit from statins. In patients with chronic liver disease and atherosclerotic cardiovascular disease, high-statin-intensity therapy is linked to a lower risk of death. In patients with impaired liver function, rosuvastatin and pravastatin exhibit pharmacokinetics similar to baseline values.
Renal impairment [47,48]	The preference should be for statins that need minimal renal elimination as the glomerular filtration rate (eGFR) decreases. In stages 4-5 of chronic kidney disease (CKD), atorvastatin is the recommended statin. Adults with stages 1 and 2 of CKD are advised to get statin medication; stages 3 through 5 should receive a combination of statin and ezetimibe. Elevated dosages of ezetimibe (10 mg) and fluvastatin (20 mg) can stop proteinuria and cause eGFR to decrease. Patients with stage 4 CKD need to have their statin dosage adjusted.
Pregnancy [49]	Generally, statins should not be used during pregnancy. It is recommended that pregnant women who are at high risk of cardiovascular disease (CVD) use statins cautiously.
Breastfeeding [50]	Because statins may cause problems for the nursing infant's lipid metabolism, they should not be administered during lactation. During nursing, other medications, including cholestyramine, colestipol, and colesevelam, might be safe.
Pediatric patients [51]	Moderate-to-high-intensity statin therapy is recommended for patients with familial hypercholesterolemia (FH).

Ezetimibe

For patients who don’t achieve the target contraction of LDL-C under the maximal tolerated statin dose, doctors recommend ezetimibe as a second-line medication [52]. It should be considered for patients who don’t tolerate stains at any dose [52]. Ezetimibe prevents the absorption of cholesterol at the small intestine brush border, as it is a Niemann-Pick C1-Like 1 (NPC1L1) inhibitor. Ezetimibe produces a notable change in the mean percentage of LDL-C, TC, TG, and HDL-C concentrations in the bloodstream [53]. Ezetimibe may have off-target effects in addition to decreasing cholesterol, including anti-inflammatory, anti-atherogenic, and antioxidant effects, which could further reduce the risk of CVD [54]. It is frequently used with statins, which improve LDL-C lowering and optimize lipid control, particularly in scenarios necessitating intense lipid-lowering strategies [9]. The combination of ezetimibe and rosuvastatin is approved by the FDA [55]. These exert synergistic action when statins exert their lipid-lowering action by reducing endogenous cholesterol synthesis in the liver. The body responds by increasing cholesterol absorption, which in turn can decrease the efficacy of statins. Therefore, the addition of ezetimibe can provide additional benefits by blocking the absorption of cholesterol, thus improving the ability of statins to reduce LDL-C [55]. Most patients suffering from cardiovascular disease or hyperlipidemia should use ezetimibe before PCSK9 inhibitors [55].

Ezetimibe can also be combined with other non-statin therapies for the treatment of hyperlipidemia. Ezetimibe has four to 12 hours as time to peak plasma concentration (Tmax), 99.7% plasma protein binding capacity, and a 22-hour half-life (t1/2), and it undergoes the metabolization process in the liver and small intestine by glucuronide conjugation [56] (Table 2). Ezetimibe causes some common adverse events, such as diarrhea, arthralgia, and upper respiratory tract symptoms [57].

**Table 2:** Summary of the pharmacokinetics of ezetimibe This table is original and created by the article authors.

Category	Pharmacokinetics
Absorption [58]	After being taken orally, ezetimibe is absorbed and converted to ezetimibe-glucuronide. Within four to 12 hours, mean peak plasma concentrations are reached. High-fat meal consumption increases the Cmax of ezetimibe by 38%. One can administer ezetimibe without taking food into account.
Distribution [59]	Ezetimibe and ezetimibe-glucuronide complexes are more than 90% protein-bound.
Metabolism [60]	In humans, ezetimibe is quickly converted to ezetimibe-glucuronide. Multiple peaks are seen in plasma concentration-time profiles, which may indicate enterohepatic recycling. CYP450 metabolizes ezetimibe.
Excretion [59]	Ezetimibe and ezetimibe-glucuronide both have a half-life of roughly 22 hours before being removed from plasma. While ezetimibe-glucuronide is expelled in urine, ezetimibe is mainly eliminated through feces.

PCSK9 Inhibitors

Hepatocytes produce PCSK9, which binds to the receptors of LDL to internalize them in hepatocytes, leading to LDL receptor degradation by the lysosomes [28]. So, this leads to a lowering of the amount of LDL receptors that are used to transport LDL-C to hepatocytes from the bloodstream [28]. Multiple approaches were tried to inactivate PCSK9 to keep the receptors of LDL and reduce LDL-C concentration in the bloodstream; the most effective one was monoclonal antibody usage [10]. 

PCSK9 inhibitors (monoclonal antibodies) inactivate PCSK9 protease by destroying the receptors of LDL. Depending on the available data, PCSK9 inhibitors have no drug interactions that have been known till now [61]. The two approved PCSK9 inhibitors are alirocumab (approved in July 2015) and evolocumab (approved in August 2015) [10]. 

PCSK9 inhibitors are a potentially useful therapy option for people whose LDL-C levels persist after high statin doses, such as patients suffering from CKD. A systematic review has been conducted to investigate the safety of biological agents, specifically in individuals with different degrees of compromised kidney function, and how well these agents correlate to lower LDL-C levels and lower cardiovascular risks in these patients. It was discovered that PCSK9 inhibitors are a dependable, secure, and effective treatment of dyslipidemia in CKD patients to lower LDL-C levels. However, since additional factors like infections increase morbidity and death, its safety and effectiveness in treating severe and end-stage kidney disease are murky [62].

With alirocumab, the major side effects are nasopharyngitis, urinary tract infection, injection site reactions, diarrhea, sinusitis, bronchitis, contusion, muscle spasms, and musculoskeletal pain [10]. The elevation of liver enzymes and allergic reactions lead to discontinuation of the drug [10]. With evolocumab, nausea, infection of the upper respiratory tract, nasopharyngitis, and back pain are the most common side effects [10]. Myalgia, nausea, and dizziness lead to drug discontinuation. In patients with serious hypersensitivity reactions that require hospitalization, PCSK9 inhibitors are contraindicated [10]. 

Fibrates

Fibrates (derivatives of fibric acid) such as fenofibrate, ciprofibrate, gemfibrozil, and bezafibrate were first developed based on the discovery of phenylethyl acetate, which could reduce serum lipid, in the 1950s [63]. Fibrates act as peroxisome proliferator-activated receptor α (PPAR-α) agonists, reducing the generation of TG in the liver [64]. Their utility is particularly notable for individuals suffering from elevated concentrations of TG and diminished HDL-C.

The most effective medications for reducing triglycerides are fibrates. They also increase HDL while lowering LDL. They are only indicated in children with hypertriglyceridemia of >500 mg/dl or at risk of pancreatitis who are not responding to dietary measures. They are preferred in cases of hypertriglyceridemia, although their use in those under the age of 18 is not yet established [65]. Unfavorable medication responses include gastrointestinal issues, rash, lightheadedness, and momentary elevations in alkaline phosphatase and transaminase levels [66]. Fibrates can lead to the formation of tumors in the long run [67]. 

Omega-3 Fatty Acids

Adults who suffer from severe hypertriglyceridemia use OM3FAs with diet modification. The exact OM3FAs’ mechanism of action is still unknown [68]. It may reduce triglyceride levels by modulating hepatic triglyceride synthesis and enhancing triglyceride clearance mechanisms [11,68]. FDA-approved OM3FA drugs are Epanova and Omtryg [68]. Omega-3 fatty acids have a cardioprotective effect by decreasing the levels of apolipoprotein CIII, which causes a reduction in the inflammatory changes related to atherosclerosis [68]. Omega-3 fatty acids also lowered the levels of non-HDL-C, TG, TC, and very low-density lipoprotein cholesterol (VLDL) [69,70]. Unlike other lipid-lowering drugs, OM3FAs do not cause any significant known clinical drug-drug interactions with other lipid-lowering drugs or alter the function of the liver [68]. 

Bile Acid Sequestrants

They prevent the reabsorption of bile acid in the ileum, which results in lowering the LDL-C levels [71]. Their use is limited due to the large number of pills, multiple drug-drug interactions, gastrointestinal side effects, and the need for suspensions [71]. They are recommended as they are the only non-statin FDA-approved drug for adolescents and children [72]. The only drug that is non-statin and approved for FH in children over 10 years old is colesevelam. FDA-approved bile acid sequestrants are colestipol, cholestyramine, and colesevelam [72].

Rosenstock et al. and Rosenson et al. reported that colesevelam lowered LDL-C, ApoB, non-HDL-C, and TG levels significantly when compared to placebo [73,74]. The adverse events of bile acid sequestrants include bloating, constipation, nausea, and abdominal pain [75]. Colesevelam, compared to colestipol and cholestyramine, has fewer drug interactions [75]. There is no necessity for the adjustment of drug doses for patients suffering from mild to moderate hepatic or renal impairment who are treated with colesevelam [75].

Bempedoic Acid

It is a novel lipid-lowering drug that suppresses the adenosine triphosphate citrate lyase (ACL) enzyme [57]. Like statins, bempedoic acid acts by blocking the metabolic pathway of cholesterol biosynthesis [76]. It is well absorbed orally and takes 3.5 hours to reach its maximal plasma concentration, and due to its long half-life (21 hours), it can be taken once daily [77]. When used with their current lipid-lowering medication, bempedoic acid may be useful for patients suffering from FH [78].

Unlike statins, bempedoic acid doesn’t cause myopathy because it is delivered as a prodrug and converts into its active form in the liver only [76]. Patients taking bempedoic acid and suffering from mild to moderate liver dysfunction don’t have to adjust their dose [77]. Food doesn’t affect the oral bioavailability of bempedoic acid [76].

Combinations of Lipid-Lowering Agents

Clinical guidelines for dyslipidemia management offer structured frameworks to inform therapeutic decisions and optimize patient outcomes. As a cornerstone in providing evidence-based care, the following synopsis delineates key recommendations from prominent clinical guidelines, providing clinicians with a comprehensive overview of best practices in dyslipidemia management.

The European Society of Cardiology and European Atherosclerosis Society Guidelines advocate utilizing high-intensity statins at the maximum dose that the patient can tolerate as a first-line treatment to achieve target LDL-C levels corresponding to the specific risk profile. If the target LDL-C values are not reached, ezetimibe-based combinations are recommended. PCSK9 inhibitors are strongly advised for secondary prevention and high-risk patients with FH. They may be added to statin plus ezetimibe in cases where they are still insufficient, especially in very high-risk patients for primary prevention. If statin-based regimens are not tolerated at any dose, ezetimibe and PCSK9i are suggested substitutes [25].

The American College of Cardiology (ACC) and American Heart Association (AHA) Guidelines, dating back to 2013, provide evidence-based recommendations for treating dyslipidemia and risk assessment [79]. The 2016 ACC Expert Consensus Decision Pathway focuses on the role of non-statin therapies, particularly ezetimibe, as adjuncts to statin therapy in higher-risk patients [80]. Furthermore, the 2017 Focused Update addresses the incorporation of PCSK9 monoclonal antibodies into clinical practice, based on cardiovascular outcomes data from the Further Cardiovascular Outcomes Research with PCSK9 Inhibition in Subjects with Elevated Risk (FOURIER) trial and the Odyssey Outcomes trial [81].

The International Atherosclerosis Society (IAS) Guidelines identified non-HDL-C as one of the major atherogenic lipoproteins and distinguished the primary strategies from the secondary ones [82]. Primary prevention prioritizes lifestyle changes, with pharmacological intervention described only for high-risk patients. Secondary prevention places more focus on cholesterol-lowering medications to attain optimal levels of atherogenic lipoproteins. Because baseline population risk varies throughout countries, risk assessment is customized to lifetime risk prediction [83].

Under the National Lipid Association Guidelines, consensus recommendations prioritize informed clinical judgment, recognizing evidence complexities, and patient involvement in treatment decisions. Recommendations align with evidence supporting the reduction of atherogenic cholesterol levels, particularly LDL-C and non-HDL-C, to mitigate atherosclerotic CVD risk [84]. 

Adherence to current clinical guidelines is imperative for navigating the complexities of dyslipidemia management, facilitating evidence-based decision-making, and optimizing patient care. By incorporating the latest evidence and expert consensus recommendations, clinicians can tailor treatment regimens to individual patient needs, ultimately reducing the burden of cardiovascular morbidity and mortality.

Future directions and emerging therapies

The landscape of dyslipidemia management continues to advance, driven by ongoing research efforts and technological innovations. While lifestyle modifications play a significant role in managing lipid-related CVD risk factors, optimal lipid control often necessitates additional interventions [85]. Numerous new targets for lipid-lowering medication have been found by recent human genetic studies, including ATP citrate lyase, apolipoprotein V, angiopoietin-like proteins 3 and 4, and apolipoprotein C-III [86]. These discoveries, combined with advancements in biotechnology, offer promising avenues for managing LDL and other lipid-related risks.

Also, although still in the preclinical animal stages, the current research investigates substitute methods for PCSK9 inhibition, including lerodalcibep, CRISPR-Cas9 gene editing, and vaccination-like tactics [87]. Lerodalcibep (formerly LIB003) represents a recombinant fusion protein comprising a PCSK9-binding domain and human serum albumin, extending its half-life to 12-15 days and enabling monthly administration via small-volume injections. Advancements in lipidomics and precision medicine herald the emergence of targeted lipid therapies tailored to individualized lipid profile characteristics and genetic predispositions. Novel therapeutic modalities, such as antisense oligonucleotide therapies targeting specific lipid metabolism pathways and gene editing techniques, offer the prospect of precise modulation of lipid levels, thereby enabling personalized treatment strategies optimized for efficacy and safety [85].

Emerging evidence implicates the gut microbiota in modulating lipid metabolism and cardiovascular risk, offering a novel therapeutic target for dyslipidemia management. Probiotics, prebiotics, and fecal microbiota transplantation represent potential avenues for modulating gut microbiota composition and function, thereby exerting beneficial effects on lipid profiles and cardiovascular outcomes [88]. Moreover, gene therapy holds promise as a transformative approach for addressing genetic forms of dyslipidemia, such as FH, by correcting underlying genetic defects and restoring physiological lipid homeostasis. Innovative gene editing techniques, including CRISPR-Cas9 technology, offer the potential for precise gene modification and long-term therapeutic efficacy, thereby offering hope for individuals with refractory hypercholesterolemia [89].

## Conclusions

Statins remain pivotal in reducing LDL-C levels and mitigating cardiovascular risk, with high-dose regimens demonstrating efficacy in patients with heterozygous familial hypercholesterolemia. PCSK9 inhibitors offer a promising adjunct to statin therapy, particularly in individuals with inadequate LDL-C control or statin intolerance. Moreover, ezetimibe serves as a valuable second-line option for further LDL-C reduction. Fibrates, OM3FAs, bile acid sequestrants, and bempedoic acid are additional therapeutic modalities, each offering unique benefits to specific patient populations. 

As the landscape of dyslipidemia care continues to evolve, adherence to current clinical guidelines and engagement in evidence-based decision-making will remain paramount to achieving optimal cardiovascular health and reducing the burden of atherosclerotic CVD. By embracing innovative therapies and leveraging the latest scientific discoveries, clinicians can pave the way for a future marked by enhanced lipid control and improved patient outcomes.
